# Optimized multi-shot imaging inspection design

**DOI:** 10.1098/rspa.2017.0319

**Published:** 2018-08-29

**Authors:** N. Brierley, C. Bellon, B. Lazaro Toralles

**Affiliations:** 1 The Manufacturing Technology Centre, Pilot Way, Ansty Business Park, Coventry, CV7 9JU, UK; 2 Federal Materials Testing Institute/Bundesanstalt für Materialforschung und -prüfung (BAM), Unter den Eichen 87, 12205 Berlin, Germany

**Keywords:** inspection design and planning, optimization, radiography, simulation

## Abstract

The inspection of complex-shaped components, such as those enabled by additive manufacturing, is a major challenge in industrial quality assurance. A frequently adopted approach to volumetric non-destructive evaluation is X-ray computed tomography, but this has major drawbacks. Two-dimensional radiography can overcome some of these problems, but does not generally provide an inspection that is as capable. Moreover, designing a detailed inspection for a complex-shaped component is a labour-intensive task, requiring significant expert input. In response, a computational framework for optimizing the data acquisition for an image-based inspection modality has been devised. The initial objective is to advance the capabilities of radiography, but the algorithm is, in principle, also applicable to alternative types of imaging. The algorithm exploits available prior information about the inspection and simulations of the inspection modality to allow the determination of the optimal inspection configuration, including specifically component poses with respect to the imaging system. As an intermediate output, spatial maps of inspection performance are computed, for understanding spatially varying limits of detection. Key areas of innovation concern the defect detectability evaluation for arbitrarily complex indications and the creation of an application-specific optimization algorithm. Initial trials of the algorithm are presented, with good results.

## 1. Introduction

Non-destructive evaluation (NDE) to confirm the absence of significant structural defects is a crucial element of quality control for safety-critical components, for example, in the aerospace and power generation sectors. A significant current industrial challenge is caused by the need to inspect components of highly complex geometries, driven to a large extent by the design freedom offered by additive manufacturing techniques. Concerns about quality assurance have the potential to impede the uptake of these novel manufacturing approaches as they transition from rapid prototyping applications to serial production of safety-critical components [1]. At the same time, it should be acknowledged that a high level of geometric complexity and the lack of an intermediate manufacturing stage during which inspection could be performed more easily (as would be the case for a subtractive manufacturing route [2]) also applies to some more conventional production processes such as casting [3].

At present, X-ray computed tomography (CT) is frequently relied on for volumetric inspection of complex-shaped additively manufactured components, because it not only provides the high level of performance independence from sample geometry and surface finish of a radiographic technique, but also gives detailed three-dimensional positional and sizing information [4]. Moreover, CT can be used to obtain dimensional measurements [5]. However, there are multiple limitations associated with CT, including the typically high per-part cost (primarily due to high equipment costs and extended cycle times) and the geometric limitations associated with the need to acquire projections from many positions along a circular arc. CT is also not fully established as an industrial inspection technique, for example, there is no personnel certification scheme as for other NDE methods. Additionally, there is an interest in having a more targeted inspection capability, which is able to incorporate prior knowledge about the component, for example, to exploit data acquired by monitoring systems on the build machine [6].

An alternative technique that, in principle, is similarly applicable to the volumetric inspection of complex-shaped parts is two-dimensional (2D) radiography. Moreover, it is a less expensive, more flexible and better established alternative to CT. However, 2D radiography does not generally provide an inspection that is as comprehensive or capable [7], most obviously given the lack of depth information in the direction of the X-rays. Additionally, designing a detailed inspection for a complex-shaped component is currently a labour-intensive task, requiring significant input from a technique expert (with American Society for Nondestructive Testing/British Institute of Non-Destructive Testing *Level 3* certification [8–10]). While there are simulation tools available to assist in this task [11–13], the level of effort required is incompatible with short production runs and part customization of the sort made increasingly cost-effective by additive manufacturing [14]. Moreover, even an experienced inspector is unlikely to be able to incorporate all available information into the inspection design, given in particular the complexity of spatially varying inspection capabilities and requirements.

The overarching aim of this work then is to improve the capabilities of multi-shot 2D radiographic imaging by computationally incorporating prior knowledge about the inspection into the inspection design, to maximize the likelihood of delivering the required level of quality assurance. The framework developed is applicable to all image-based inspection modalities (subject to the provision of a modality-specific inspection simulation capability), but exemplified using 2D radiography—the extension to surface inspection at optical wavelengths is also likely to be of interest for the inspection of samples of high geometric complexity. As an intermediate output, the algorithm computes spatial maps of inspection performance, an output that is itself of great potential value for understanding the limitations of an inspection, for example by facilitating the extrapolation of limited experimental data. Central to the algorithm is the capability to quantify the value of an inspection configuration with respect to the quality assurance requirements, and a numerical optimization algorithm with features designed for the application described.

The work is complementary to that described in [15], which relates to the optimal selection of radiographic projections for subsequent tomographic reconstruction, rather than 2D imaging. Key differences between that work and the present study, other than the application, relate to the choice of the means of quantifying the value of a projection and the nature of the applied numerical optimization: the greedy sequential scheme, based on exhaustive enumeration, that Fischer *et al*. use would incur an overwhelming computational cost in higher dimensional decision variable spaces (as examined here), and has a low likelihood of converging to the overall global optimum.

The paper proceeds by setting out some of the theory underlying the framework developed. Section 3 then provides an overview of the different components of the framework, while §§4 and 5 focus on the two primary areas of innovation, the detectability evaluation and the optimization, respectively. Section 6 provides details of initial computational and experimental trials conducted, and discussion. The conclusion section rounds off the paper.

## 2. Theoretical background

### (a) Operating principles

The algorithm developed seeks to incorporate all prior knowledge available about the specimen and equipment into the inspection design. This most importantly includes knowledge about the component geometry and material, but can also extend to details about expected defect types and critical regions, as provided by stress calculations. The ultimate objective is to be able to compute the set of radiographic projections that in total provides the optimal inspection performance within operational constraints (such as hardware limitations and permissible inspection time). While skilled human inspectors can design inspections trying to incorporate such prior knowledge into the inspection procedure, it is expected that for highly complex components and detailed *a priori* information, such planning is unlikely to yield even near-optimal performance, and the limitations of the design chosen are unlikely to be well understood without the use of computational aids.

**Figure 1.**
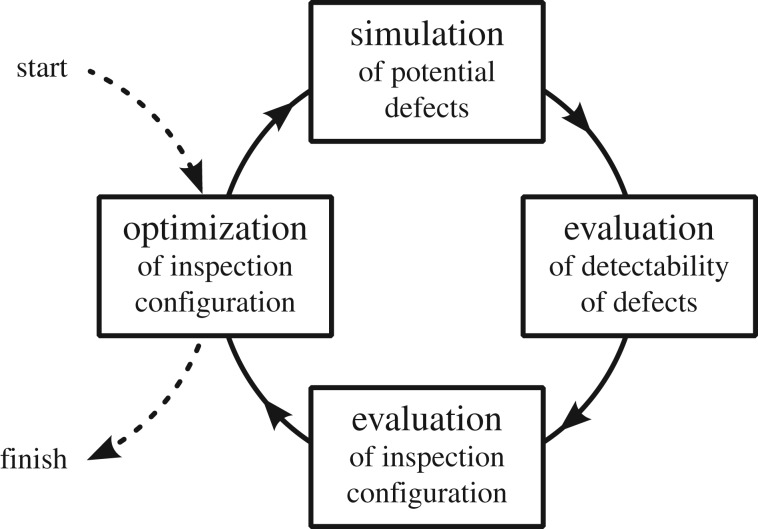
Schematic of the process underlying the algorithm. In the implementation developed, the element that is most specific to the inspection modality studied, the simulation, is delegated to a discrete software package controlled externally.

At the heart of the algorithm is a computer simulation of the inspection. This provides the *forward model*: for a given inspection configuration an expected output can be calculated. Importantly, for a given hypothetical defect, the indication that will be present in the inspection data can be predicted. This indication can then be evaluated to assess the ability of that inspection configuration to provide useful information about that hypothetical defect. Then by sampling many hypothetical defects, informed by available prior knowledge, a broader understanding of that inspection configuration’s capabilities can be obtained. This can then be optimized within the constraints provided by e.g. the available hardware, to find the inspection configuration that provides the maximal inspection performance. This process, illustrated in figure 1, is an example of *inversion*: finding the input required for a given desired output.

### (b) Key assumptions

As with every model, there are some key assumptions that underlie the approach taken in the algorithm developed, and it is important to understand these so as to be aware of the model limitations. The most important assumptions identified are discussed below.

#### (i) Uncalibrated model

The simulation model used in this project is uncalibrated. This means that there are multiple parameter values that are expected to deviate both from those nominally set on the inspection hardware, and from those that would give the model output closest to the experimental output. An immediate consequence of this is that the program developed is unable to provide any information on absolute performance (defect detectable versus not detectable in this configuration), only relative performance (defect more detectable in this configuration than the last). Additionally, it is assumed that the parameters that are mismatched between simulation and physical experiment have negligible impact on the detectability associated with the parameters that are being optimized over—the two sets of parameters must not exhibit any correlation in detectability. This is a reasonable assumption for the case of the radiographic inspection studied, as for example, the exact X-ray source spectrum (described by uncalibrated parameters) is unlikely to affect the choice of sample positioning (described by parameters optimized over) for optimal detectability. However, model calibration, for example building on [16], is an obvious example of suggested further work.

#### (ii) Definition of objective function

Every mathematical optimization is only as useful as the objective function it is seeking to maximize/minimize is meaningful in practice. A great deal of thought has gone into the definition of the objective function used in this algorithm, as will be described further in §3f. It does however also incorporate a measure of user choice, so there is no guarantee that this will be appropriate under all circumstances and for all users, and should be modified to suit different user requirements where necessary.

#### (iii) Sampling

The objective function in this algorithm is evaluated by the sampling of hypothetical defects. Inevitably, the more defects that are sampled, the more accurate the computations, but the longer these will take. This is in effect a *Monte Carlo* integration approach, so in line with general theory a $1/\sqrt{N}$ improvement in the model accuracy with *N*, the number of samples taken, may be expected [17]. However, in addition to this mathematical effect, one must be aware that the choices/assumptions underlying the sampling must be appropriate to give a useful output. This, for example, relates to the possible choice to bias the spatial sampling of defects to a particular region of the component volume, in an effort to compute a targeted inspection design, which may be appropriate if that region is considered likely to contain defects (e.g. based on historic data), but may be inappropriate if that same region is also least critical (from lifing calculations).

#### (iv) Local optimum

The complexity of the optimization in this work is especially dependent on the geometric complexity of the sample studied but, in general, can be expected to be high. In most applications, the objective function space that the optimization explores can be expected to be characterized by a high dimensionality and many local optima. This means an appropriate global optimization algorithm must be applied, but even with such a tool, there is no guarantee that the algorithm will converge to the true global optimum in any reasonable computation time. Moreover, as exhaustive enumeration of the objective function space is out of the question, the true global optimum may never be known, and the best option for obtaining a degree of confidence in the point reached by the optimization is likely to be based on re-running the optimization many times from different starting positions and/or with different random seeds (given the stochastic nature of most global optimization algorithms). In short, without excessive computational effort it is impossible to be sure that the point converged to represents the very best configuration conceivably possible, but the point should at least represent a reasonable choice of configuration. This does in practice also mean that if a ‘reasonable guess’ of a good inspection configuration is available, it is likely to be worthwhile to initiate the optimization from this. Details of the optimization are described in §5.

#### (v) Computational limitations

The work makes some assumptions about computational constraints. Specifically, it is assumed that for the algorithm to be useful industrially, reasonable results must be obtainable within a week of running on a high-performance, but stand-alone, computer. However, evaluation speed is not a direct concern at present as in practice any algorithm prototype can be accelerated using (continually improving) more advanced hardware and re-writing the code for speed specifically. Nonetheless, for development purposes it is ultimately necessary for the algorithm to be capable of returning useful results in a time-frame measured in minutes and hours rather than days. Thus, the likelihood of converging to a local optimum is further heightened.

## 3. Method overview

This section seeks to provide an overview of the framework developed, but the two elements that this paper focuses on, the detectability evaluation and the optimization, are described in the dedicated §§4 and 5, respectively.

### (a) Algorithm architecture

The framework architecture is based on that developed in [18]. This lends itself well to the development of an optimization framework thanks to the inherently flexible and extensible design, as well as the incorporation of tools for efficient computation. The linear sequence of modules that makes up the framework are described (in order) in the sections that follow: the inputs available to a given module are specified by the outputs of the preceding modules.

### (b) Simulation

The *forward model* is provided by the aRTist radiographic simulator software package [11,19]. This software tool is able to predict the indication to be expected from a given hypothetical defect in a specific sample geometry obtainable in a specific inspection configuration. The following features were provided or enabled specifically for this work:

— Remote control: allowing the simulation to be controlled from an external script;
— CAD voxelization: enabling a CAD-model to be converted into a voxel dataset of a user-defined coarseness; and
— ROI evaluation: allowing the image evaluation to be restricted to a region of interest (ROI) subset automatically determined from the inspection scene configuration.

Note that the alternative simulation packages CIVA RT [12] and XRSIM [13,20] were considered, but these were determined to be unsuitable as they have no interface to enable external control. The latter in fact already offers a built-in capability for mapping X-ray inspection coverage, however no details on the workings of the software are published, so robust comparisons are impossible. Nonetheless, it is clear that XRSIM for instance does not provide any capability for numerical optimization of the inspection design of the sort presented here. For the application of the optimization framework to an alternative (non-radiographic) inspection modality, the simulation module would need to be exchanged for an appropriate modelling capability.

### (c) Detectability evaluation

This module computes one or more metrics describing numerically the detectability of a defect indication seen in radiographic images—in practice an area–contrast to noise ratio (CNR) relationship for each indication. This is described in detail in §4.

### (d) Defect sampling

**Figure 2.**
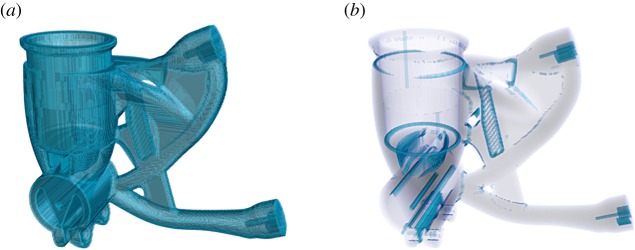
Renderings of two possible permissible regions for defect sampling calculated from the voxelization of a component’s CAD-model (see §6b). (*a*) The region appropriate for sampling near-surface defects, while (*b*), a region appropriate for sampling trapped-powder-type defects. The voxels here are cubes of 0.5 mm edge length. (Online version in colour.)

Morphological operations (erode and dilate) on the binary array that is the voxelized representation of the sample geometry allow the calculation of the permissible region for different defect types as specified by the user: surface defects, internal defects and traces of the manufacturing process, such as trapped powder in the case of additive layer manufacturing. Illustrations of such permissible regions are provided in figure 2.

**Figure 3.**
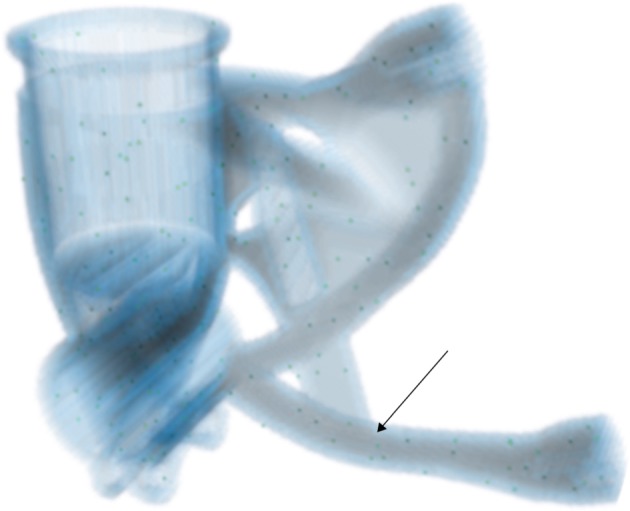
A rendering of the permissible region computed for a component’s CAD-model (see §6b) appropriate for sub-surface defects, with the chosen defect sampling locations superimposed for the case of 200 defect samples. The arrow relates to §6c. (Online version in colour.)

The user can provide prior knowledge about the spatial distribution of the defects the inspection is to be steered towards. This allows the incorporation of *zoning* conventionally applied to complex components. In addition to the defect locations, multiple other defect properties can be sampled over, if there is prior knowledge to justify this. Therefore, defect aspect ratios and orientations, for example, can be specified in terms of statistical distributions to be sampled, to describe the approximate morphologies and expected alignments of defects, respectively. Moreover, it is possible to specify different defect types independently, including their relative significance. All quantities to be sampled over are then sampled using a *Sobol* sequence, an example of a *low discrepancy sequence*, for optimal space-filling properties and hence consistent *Monte Carlo* performance [17]. Such pseudo-random sequences have the further advantage over alternative approaches that they allow for the sample set to be extended after creation, so potentially the algorithm could iteratively refine the sampling performance. An illustration of the spatial sampling is provided in figure 3.

### (e) Projection sampling (decision variable specification)

The sampling of (radiographic) projections that make up a projection set and describe an inspection configuration is controlled by the optimization algorithm (see §5), as it is this set of parameters that the algorithm seeks to compute—the decision variables/optimization parameter space. However, the user must provide the guidelines within which this optimization is to take place. This includes the number of projections in the set, which associated parameters should be varied and within what ranges (optimization constraints), as well as links between parameters of projections in the set.

The last point relates to the fact that it is possible to link parameters across the projections of a set. For example, this means that while all three *Euler* angles describing the sample orientation may be optimized over for all projections in the set, the values of two of these for all projections in the set must be the same—as exemplified in table 1. This is attractive not only as it reduces the dimensionality of the parameter space being explored by the optimization (and thus potentially the computation time, for a given coarseness of space exploration), but also means the same static fixture can be used for all projections when acquiring the data using an X-ray system with a single rotational degree of freedom—as in most industrial CT machines.

**Table 1.** Table illustrating the 14 decision variables for an example set of three projections with two linked rotational parameters (as considered in 6f). The two shared rotational parameters, *ρ* and *ϕ*, rotations about *x*- and *z*-axes, respectively, can be thought of as specifying the fixture to be used in an X-ray system with a single rotational degree of freedom (about *y*-axis).

	projection 1	projection 2	projection 3
translational	*x*_1_	*x*_2_	*x*_3_
	*y*_1_	*y*_2_	*y*_3_
	*z*_1_	*z*_2_	*z*_3_
	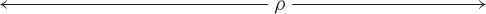		
	*θ*_1_	*θ*_2_	*θ*_3_
	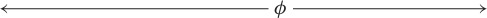		

### (f) Objective function

In the current implementation of the algorithm, the objective function that the optimization seeks to maximize is computed for a given inspection configuration and defect sample set by the steps outlined below. As emphasized in §2b, there are multiple valid alternatives.

(i) The area–CNR relationship determined for each defect indication is turned into a single metric of detectability using the functional form described in §4.
(ii) Across projections within the projection set describing the inspection configuration, the maximum detectability metric is computed for all defects sampled.
(iii) Among the maximum detectabilities, the 20th percentile is determined, interpolating as required. The mean of that value and all detectability values falling below it is then evaluated.

Thus the optimization will maximize the lowest fifth of the maximum detectabilities across the projections. The particular implementation of the objective function evaluation is designed to both be robust to outliers and avoid unnecessary discontinuities that would only add to the complexity of the optimization task.

### (g) Optimization initialization

*Latin hypercube* sampling is used to provide the initial population of the evolutionary algorithm described in §5. This sampling strategy is preferred here over generic random sampling for reliable space-filling properties and chosen over the use of a *low discrepancy sequence* (see §3d) as here the number of samples required is fixed, without the possibility of needing to extend the set of samples [21].

### (h) Optimization

This module completes the search for the parameter combination describing a projection set that offers ‘the best’ objective function value—see §5.

## 4. Detectability evaluation

**Figure 4.**
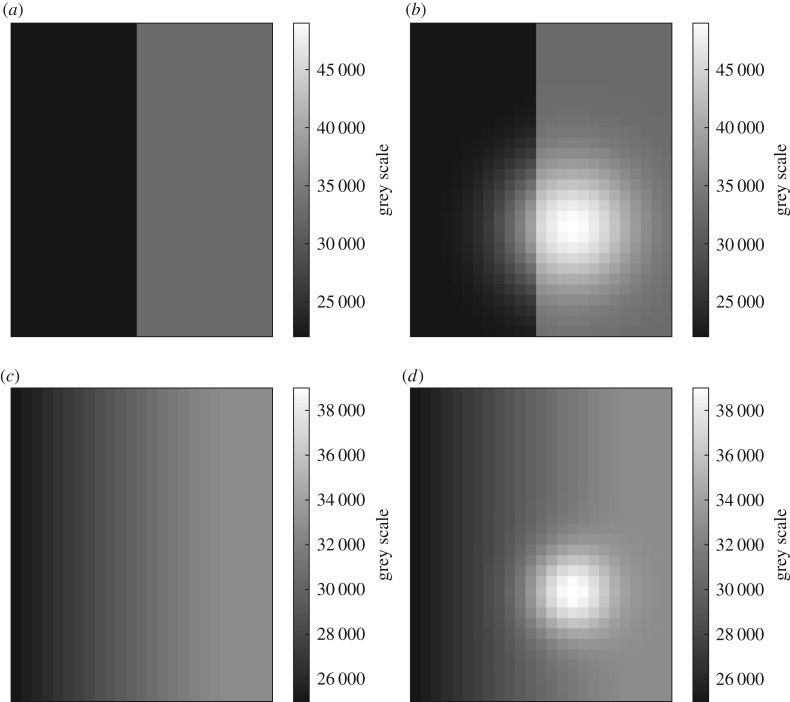
Artificial example baseline and indication images, that the detectability evaluation must be able to process. (*a*) The case of a hard edge, (*b*) the corresponding example indication, while (*c*) the case of a background with a gradient and (*d*) a corresponding example indication image.

For each defect sampled the simulation provides an image of the simulated indication, as well as an image of the same region in the radiograph if there is no defect present—the baseline image. Examples are provided in figure 4.

These two images must then be processed to obtain (one or more) numerical values that represent the detectability of the defect in question. The detectability computation should be broadly consistent with human visual inspection, even if the evaluation is, in practice, automated [22]. The approach taken acknowledges that the ability to distinguish a patch of a different greyscale (indication) from the noisy background is dependent both on contrast to the background noise level (CNR) as well as the area of the patch [23].

**Figure 5.**
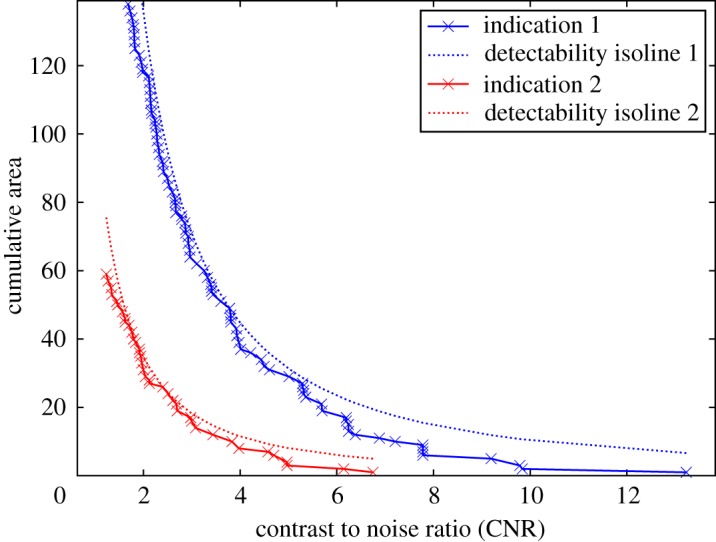
Cumulative area–CNR plots computed for the two baseline—indication images presented in figure 4. The images from figure 4*a*,*b* have been plotted as the line labelled indication 1, while the indication 2 label relates to figure 4*c*,*d*. Fitted tangential isolines of constant detectability of the functional form suggested in equation (4.1) are also shown. The values of *k* used were 20.2 and 10.3, for indications 1 and 2, respectively. (Online version in colour.)

However, in practice, indications of complex defects and/or in complex component geometries are unlikely to be characterized by a single contrast level or equivalently area. Moreover, the use of a ‘form factor’ to handle non-circular indications [24] is not general or computationally robust enough for present purposes. Therefore, here we extend the description of detectability in terms of CNR and area to a distribution. An example of this is shown in figure 5. The points on this cumulative, *survival function*, presentation give the image area that is characterized by a CNR at least as high as the value plotted. Detectability increases along both orthogonal axes so this space is similar to the objective function space of a 2D multi-objective optimization. For example note that in figure 5, the line for the first indication is consistently above and to the right of the line for the second, reflecting the overall higher contrast and larger size of the first indication versus the second, and hence indisputably higher detectability of the first indication compared with the second. In the terminology of multi-objective optimization, the points of first line *dominate* those of the second [25].

Such a distribution can meaningfully be computed for any indication, no matter how complex the defect morphology, the sample or the noise behaviour. An outline of the processing steps is provided below. The initial steps are similar to those applied in [26].

(i) Subtraction of baseline image from indication image to obtain a contrast map.
(ii) Segmentation of contrast map into indication and background pixels. Given that the inputs are from simulation, the background will in most cases be zero after subtraction, simplifying the segmentation.
(iii) Local evaluation (using a small kernel) of the noise level as the standard deviation for the indication region found by the segmentation.
(iv) Computation of the contrast to noise ratio, enforcing a maximum value to handle low noise inputs.
(v) Evaluation of pixel cumulative statistics from map of CNR, to obtain cumulative area–CNR plot.

This calculation then allows every defect evaluated in the simulation to be converted into an area–CNR table representing detectability. Ultimately, for many objective function definitions (see §3f), it is in practice desirable to condense this to a single value that can be directly compared between defects/configurations. This necessarily involves specifying a relationship between indication area and CNR that describes the trade-off between these two quantities. Here, a functional form has been chosen to describe constant isolines of detectability in the area–CNR plane. This functional form must, given the application, satisfy several key conditions: it must be monotonically decreasing in area with CNR, it must increase monotonically in area and CNR with the detectability parameter *k* and have asymptotes in both area and CNR. The general form adopted here is given in equation (4.1),

4.1 $$A=\frac{k^{2}}{C^{b}},$$

where *A* is the area, *C* the CNR, *b* a positive implementation-dependent parameter to be determined by fitting to example data (*b* = 1.6 used here) and *k* is squared such that *k* is proportional to the equivalent radius of the indication area. This functional form can then be used to determine the *k* value of the isoline that is tangential to the *survival function* plot, and hence the maximum detectability associated with that representation of the indication. This is illustrated in figure 5. This approach is believed to be novel.

## 5. Optimization

The optimization of the projection set for an arbitrary component geometry faces many challenges. For example, as stated in §2b, the objective function space that the optimization explores will, in general, be characterized by many local optima. This is a consequence of the fact that, for all but the simplest sample geometries, detectability of a defect collection is likely to vary in a complex manner with any one of the key parameters (e.g. sample angle about a vertical axis in one projection). Additionally, the objective function space may contain discontinuities and hence not be differentiable everywhere. This is caused by the discontinuous variation in material path with orientation associated with some possible sample geometries, for example. There are many global optimization algorithms that may reasonably be applied to such problems, but in general the choice of algorithm and its control parameters will need to be tuned to the specific problem for consistently good performance [27].

The algorithm adopted is a version of the genetic algorithm (specifically the (*μ* + λ) form) [28,29]. As the name implies, this mimics Darwinian evolution, seeking to find the optimal solution by evolving a population of candidate solutions through a sequence of cross-over, mutation and selection operations. The chromosomes manipulated encode the decision variables describing the varied parameters of the projection set via value encoding, but with the variables normalized to [0, 1]. The cross-over operator selected is the two-point operator [30], the mutation operator is of the Gaussian perturbation form and the selection operator a tournament selection [31].

Given the desire to converge to at least a local optimum in a reasonable space of time, the algorithm is in fact structured to be used in two stages—first an exploratory phase, featuring large mutations, for example, followed by a refinement phase, characterized by smaller scale mutations only.

Further properties of the specific optimization challenge and associated features of the novel algorithm implemented are detailed in the subsections that follow.

### (a) High-dimensional—and constrained

The parameter space to be explored is likely to be high-dimensional, as a consequence of having many parameters that describe an individual projection, and even more that describe a projection set, many of which a user is likely to want to try to solve for. This property does not, in principle, make the objective function evaluations more complex, but does mean a greater number of samples is required to explore the search space to an appropriate extent—thereby incurring a computational penalty. Additionally, all the parameters optimized over are constrained to user-defined intervals (derived e.g. from hardware manipulator ranges). The constraint enforcement approach adopted in the algorithm implementation involves the use of reflective, symmetric boundaries, so points picked outside the feasible region are mapped back into it.

### (b) Expensive—but deterministic

Each projection set evaluation is relatively expensive, typically taking multiple minutes to compute. This means that it is desirable for the algorithm to select its evaluation sites with particular care to maximize the information obtainable from each one. On the other hand, the objective function evaluation is entirely deterministic (given that the same samples of hypothetical defects are used for each evaluation, assuming that all ‘random noise’ in the simulation is seeded consistently). This means that it is permissible and helpful to incorporate historic information about the progress of the optimization into the evolutionary process, not just information from the last generation evaluated. This is achieved by establishing an approximation to the underlying objective function space from the evaluations to date and feeding insights gained from this into the evolutionary process (surrogate-based optimization) via newly generated offspring. This can be thought of a supplementary mutation operation. Different surrogate surface generation approaches can be called upon in the implementation developed, including *Kriging* and radial-basis-function interpolation [32,33]. Additionally, a minimum distance in parameter space is enforced between evaluated locations to avoid duplicate evaluations.

### (c) Symmetric

The physics of the problem being investigated mean that for many applications, the objective function space will be characterized by symmetries. To avoid unnecessary computational waste, it is desirable to restrict the evaluation of the objective function space to a section that is unique. The problem of symmetries in objective function spaces is well known [34,35]. The problem is addressed by introducing one or more symmetry-breaking constraints, for instance reducing the range of a parameter with respect to which the objective function is symmetric.

The most important source of these symmetries is the physical irrelevance of the order in which projections within a set are labelled or acquired—or equivalently, the commutativity of the objective function calculation (see §3f) with respect to the projections in the set being evaluated. Note that the benefit of symmetry-breaking for the case of *r* interchangeable projections in the set being optimized over scales with *r*! (the number of equivalent permutations), so rapidly grows for large projection sets. Such symmetries from interchangeable parameters are typically broken via a lexicographic sort of the parameter values, such that only monotonically increasing parameter combinations are ever evaluated [36]. However, in the case of the optimization of projection sets, the symmetry is not between individual parameters, but between the sets of the parameters associated with different projections. There is the further complication, due to the potential linking of parameters (see §3e), that some parameters optimized over in fact relate to all projections in the set. Therefore, some development is required to implement a symmetry-breaking constraint.

The solution developed and adopted hinges on grouping the parameters optimized over by the projection they relate to, grouping separately the parameters that are shared between projections, and then ordering the parameters of each group (excluding the group of shared parameters) according to group order. This requires each group to be reduced to a single numerical value that can be used for the purpose of the lexicographic sort of groups. There are numerous viable options for the choice of (*hashing*) function for this dimensionality reduction—the only absolute requirement is that this allows a unique order of groups to be defined. However, it is desirable from the point of understanding and enabling efficient surrogate surface calculation (see previous section) for the function to behave monotonically for all possible inputs, and hence the explored region of the parameter space to be contiguous. The function chosen for the present algorithm implementation is a simple sum of the inputs. This may be supported by a lexicographic sort based on the parameters of the groups to overcome the potential problem of different inputs giving the same output (a ‘hash clash’). Note that the parameters shared between projections play no part in the symmetry being addressed, so they can simply be prefixed (or appended).

This sorting then means that the projection-order symmetry is broken and only a unique section of the objective function space is explored. This is illustrated later in figure 7*a*. An example sort is provided here, for the case of a projection set of three projections, each specified by three parameters and two parameters shared across the set:

$$\underset{\text{shared}}{\underbrace{0.9,\;0.8,\;}}\;\underset{\text{projection}\;1}{\underbrace{0.8,\;0.3,\;0.5,\;}}\;\underset{\text{projection}\;2}{\underbrace{0.3,\;0.5,\;0.8,\;}}\;\underset{\text{projection}\;3}{\underbrace{0.4,\;0.2,\;0.3}}\Rightarrow \;\underset{\text{shared}}{\underbrace{0.9,\;0.8,\;}}\;\underset{\text{projection}\;3}{\underbrace{0.4,\;0.2,\;0.3,\;}}\;\underset{\text{projection}\;2}{\underbrace{0.3,\;0.5,\;0.8,\;}}\;\underset{\text{projection}\;1}{\underbrace{0.8,\;0.3,\;0.5}}$$

### (d) Combinatorial

A further noteworthy feature of the optimization is that the optimization seeks to find a combination of projections that gives optimal performance, and the evaluations of the individual projections are essentially independent of each other. Efficient caching of intermediate results for the constituent projections of the evaluated projection sets then enables the reuse of these results to potentially generate a great number of additional projection sets, distinct from those originally sampled. This process is attractive computationally as the projection-specific calculations (featuring many indication evaluations) are the computational bottleneck, rather than the subsequent evaluation of the projection set to form the objective function (see §3f), the computational effort of which is negligible. In principle, this gives ${\;}^{n\cdot r}C_{r}-n$ additional projection sets, where *n* is the number of projection sets evaluated to date, each containing *r* projections (assuming uniqueness of projections), a very large scaling factor for many practical scenarios (e.g. 17 280 additional projections for *n* = 16, *r* = 3). These points will almost certainly greatly improve the ability of the optimization to efficiently converge to a solution at essentially no cost, and can readily be incorporated into the genetic algorithm as additional offspring, alongside those evaluated directly.

However, there is a practical complication and reason why this performance improvement will not be realized in practice: parameters shared between projections in the set. Such parameters limit the extent to which projections can be re-combined with each other to create additional projection sets at minimal cost, as they introduce a dependence of the projections in a set on each other. Re-combinations are then only permissible between projection sets which maintain the same values for the shared parameters. Given that all parameters will be varied as the genetic algorithm evolves its population of candidate solutions, one can envisage a situation where essentially each projection set evaluated features a unique set of shared parameters, completely nullifying scope for the combinatorial scaling.

**Figure 6.**
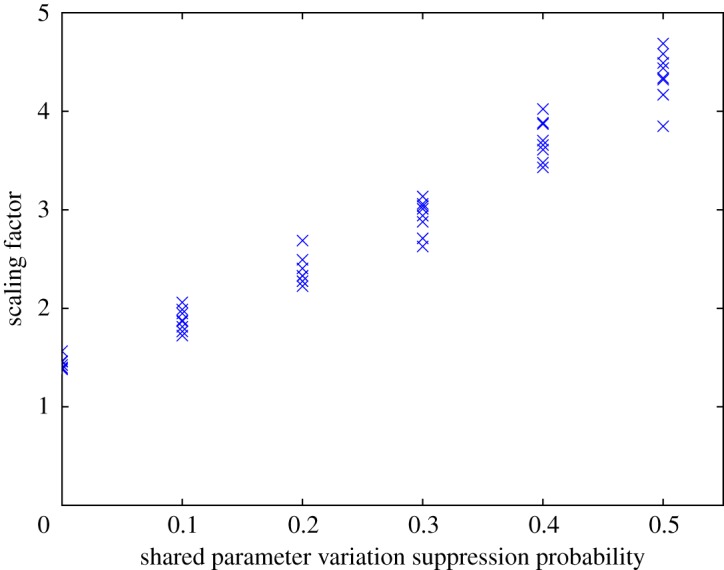
Plot of the scaling factor achieved (the total number of projection sets evaluated, including by re-combination, divided by the number directly evaluated) for different shared parameter variation suppression probabilities, for the case of a test problem with a four-dimensional parameter space consisting of two shared parameters and two projection-specific parameters, after 16 generations of 24 samples (plus 16 initial samples, so *n* = 400 and *r* = 2). At each value of the suppression probability trialled, the calculations were repeated with different seeds for the random number generator. There is a clear increasing trend in the scaling factor with suppression probability. Note the scaling factor theoretically achievable here in the absence of shared parameters would be 799. (Online version in colour.)

**Figure 7.**
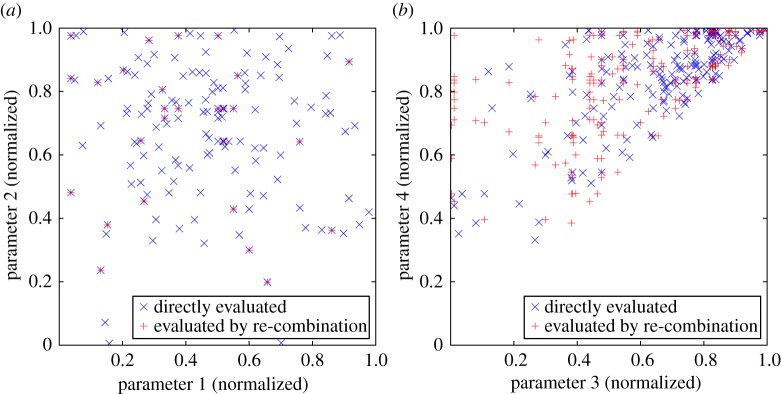
An illustration of the generation of additional projection sets for a test problem with a four-dimensional parameter space consisting of two shared parameters and two projection-specific parameters. (*a*) Relates to the two shared parameters, (*b*) to the two independent parameters. The locations evaluated over eight generations of 24 samples (plus 16 initial samples, so *n* = 208), with the shared parameter variation suppression probability set to 0.2, are plotted, distinguishing between the direct evaluations and those indirectly evaluated by re-combination of previously evaluated projections (a total of 247 points). In (*a*), the coincidence between direct and indirect evaluations is evident, in (*b*), the pattern of the indirect evaluations is revealed: these are arranged in vertical and horizontal lines with respect to the direct evaluations. Additionally, the latter plot illustrates the action of the symmetry-breaking described in §5c, as all the points lie one side of the leading diagonal through this projection of the parameter space. (Online version in colour.)

The solution devised hinges on the stochastic suppression of variations in the shared parameters via the introduction of a further algorithm control parameter that expresses the probability of such suppression occurring. Then, when suppression is invoked (for example, after a random mutation of the parameter values), the shared parameters are mapped to a set of values previously used in evaluations. As illustrated in figure 6, a non-zero control parameter value has the potential to greatly enhance the scope for additional projection sets to be evaluated, but does so at the expense of a more comprehensive exploration of the subset of the parameter space that is shared between projections. This behaviour is exemplified in figure 7*a*,*b*. The choice of the suppression probability should therefore take into account the relative dimensionality and expected importance of the shared versus projection-specific parameter sub-spaces. Initial tests suggest that enabling the evaluation of projection sets via re-combination brings substantial performance improvements over not using this option, but larger shared parameter variation suppression probabilities can readily impede the correct convergence of the global optimization algorithm.

## 6. Demonstration: results and discussion

In this section, we build up to a complex optimization trial (§6f), including a demonstration of the experimental application of the optimization outputs, based on the inputs described in the initial subsections, by first assessing individual defects (§6c), then assessing a population of defects (as expressed by the objective function) over a range of inspection configurations for a simple sample geometry (§6d), then carrying out an initial optimization on that simplified scenario (§6e). This hierarchical approach is designed to provide confidence in the operation in each level of the algorithm, and provide some context to the later subsections, given the constraints on quantitative validation imposed by the assumptions of the work (§2b).

### (a) Inputs

Given that the high level purpose of the algorithm is to incorporate prior knowledge into the inspection design to improve the performance of the inspection, the algorithm necessarily takes multiple inputs. Some of these are optional, and default values can reasonably be assumed, others mandatory. The main inputs are detailed below, grouped by type.

#### (i) Inspection description

Parameters in this class specify the inspection as far as possible. They include:

— Sample geometry—to be provided as a CAD-model
— Sample material—specifically elemental composition and density
— Specification of defects to be considered, potentially including the specification of properties such as orientation and spatial distribution.
— Specification of the projection set. This includes any parameter links across projections, the specification of which parameters to vary in the optimization and ranges for all these parameters.
— Specification of the inspection hardware available—including operating ranges for manipulators and the X-ray source

#### (ii) Computation parameters

These parameters specify the nature of the computation to be completed, specifically the user’s choice on the accuracy–computing time trade-off and the computing resources to use. Key examples of this type of parameter include the number of defect samples to process per projection evaluated and the number of generations to evolve in the optimization algorithm. Note that in all computations to date the very computationally demanding scattering computation in the simulation has been omitted—so all results presented are derived from direct radiation calculations only.

#### (iii) Control parameters

These low-level parameters defining the details of the calculation all have default values. Parameters that fall into this class include the pixel margin to be maintained around the indication by the ROI evaluation and the probability of a cross-over occurring during the execution of the genetic algorithm.

### (b) Test case description

The sample selected for the experimental demonstration is a half-scale version of an additively manufactured motorcycle front fork end. The component design has been subject to light-weighting by topological optimization, providing a component with significant geometric complexity, as seen in figures 2 and 3, ideal for this project’s purposes. The component considered here was made by laser powder bed fusion in 316L Stainless Steel. The component’s bounding box measures 7.9 × 6.9 × 2.9 cm. The sample is courtesy of GRM Consulting Ltd, K-Tech & MTC.

### (c) Example detectability computations

In this section, we consider a single defect and examine its computed variation in detectability in different projection configurations. The defect considered is 0.7 mm spherical void inserted in the geometry in the location indicated by the arrow in figure 3.

**Figure 8.**
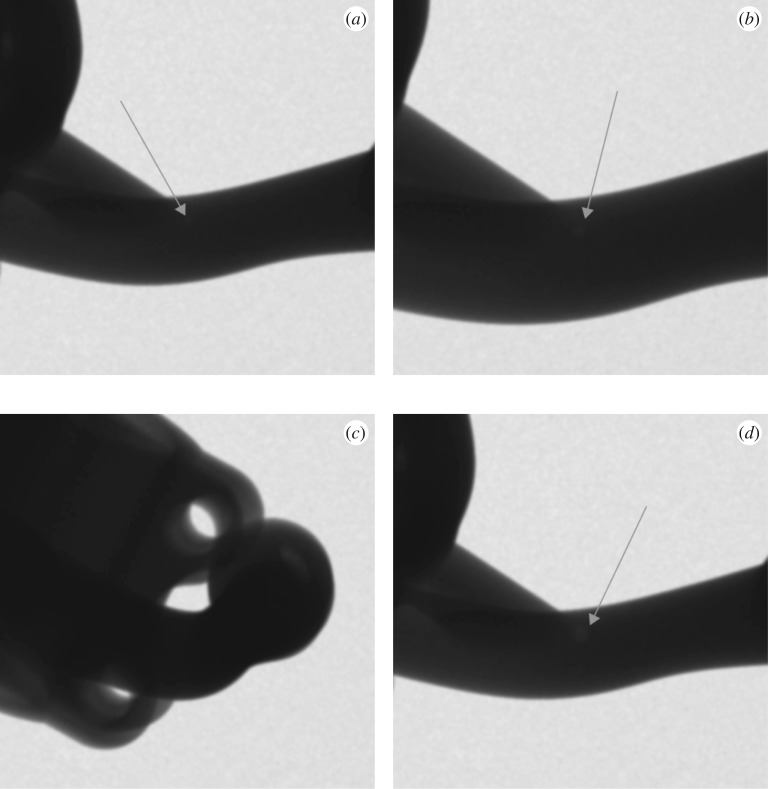
Simulated radiograph sections for the modelled pore in different configurations: (*a*) the output of initial configuration to be treated as the baseline for comparison purpose, (*b*) the effect of a higher magnification, (*c*) the effect of a configuration of longer material path compared with the initial configuration (sample rotated about the defect) and (*d*) the output when the defect size has doubled. Note that by adjusting the grey scale mapping it is readily possible to reveal indications in all these images except (*c*) in the locations of the superimposed arrows.

The simulated radiographs for the defect in different sample arrangements are shown in figure 8. There is an initial image, that the others are to be compared against, while the other images consider the effect of a higher magnification, longer material path, and larger defect size, respectively. The detectability metric values computed were 8.7, 20.4, 4.9 and 53.2, respectively. The computed detectability metric values are qualitatively consistent with what would be expected from radiographic experience.

### (d) Mapping example objective function space

Having in the preceding section examined the detectability metric assigned by the algorithm to a single defect, here we define a simple objective function space and plot this to further build confidence in the behaviour of the algorithm. For computational and illustration purposes, the objective function is a function of only two parameters. The test scenario consists of a rectangular cuboid of iron, to be inspected in a single projection, where most settings (e.g. those relating to the source and detector) are pre-determined, and in fact the only parameters to be varied are the orientation of the cuboid about the vertical axis (parallel to the median edge length) and the position of the sample along the source–detector axis (and hence magnification). The defects considered are 1 mm diameter spherical voids, and 200 such defects are uniformly sampled per projection evaluated.

**Figure 9.**
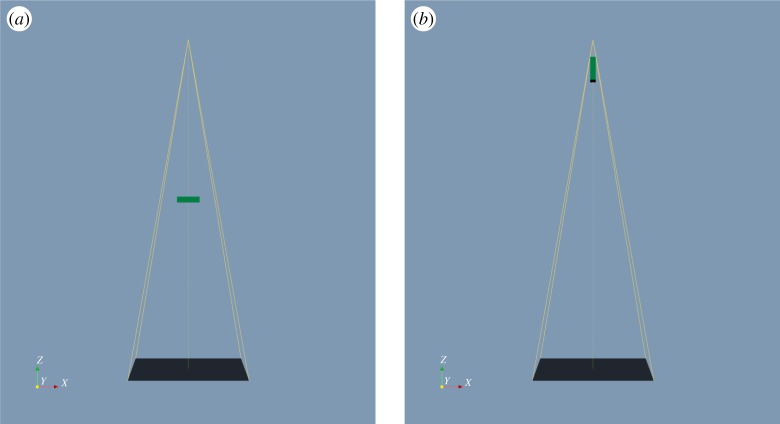
Scene views in aRTist illustrating the extremes of the 2D parameter space explored. In (*a*), the magnification and orientation angle are both at the minimum permissible value, while in (*b*), both variables are at their maximum permissible values. (Online version in colour.)

**Figure 10.**
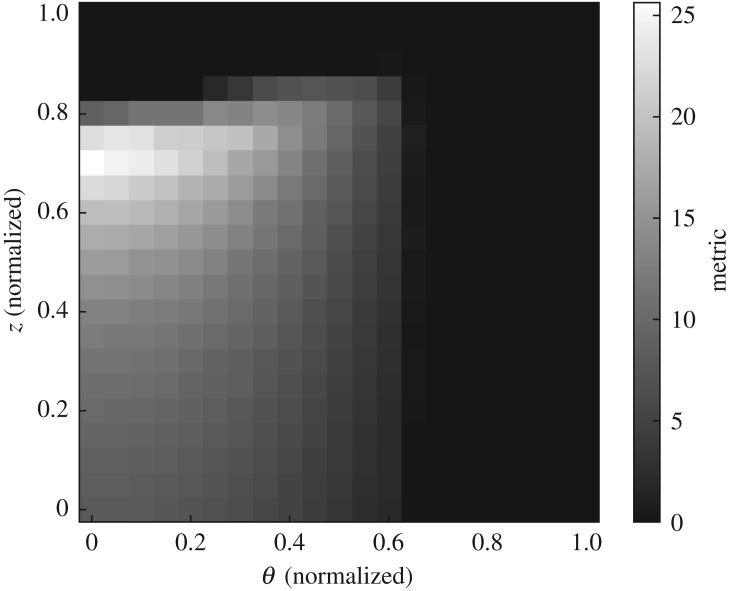
Map of the 2D objective function space associated with varying the orientation angle *θ* and the distance to the detector *z* (specifying the magnification) while holding all other variables constant. The variable axes are normalized to [0, 1], spanning the value ranges illustrated in figure 9, such that (*a*) represents point (0, 0) and (*b*) (1, 1).

Figure 9 illustrates the extremes of the parameter ranges, in angle and magnification axis position, considered. The corresponding map of the objective function space is plotted in figure 10. This plot has several interesting features. Consistent with practical radiographic experience, the objective function increases with increasing magnification, but decreases with higher sample orientation angles (due to the longer material path associated with many potential defect positions). The trends observed are reasonably smooth, giving confidence in the numerical stability of the algorithm, for example. However, the plot does reveal a large level region of zero value: both at very high magnification and at higher orientation angles the detectability metric falls to 0, as parts of the sample move out of the field of view or become occluded, respectively, such that the defects sampled in those sections of the specimen are entirely undetectable.

### (e) Initial optimization test

**Figure 11.**
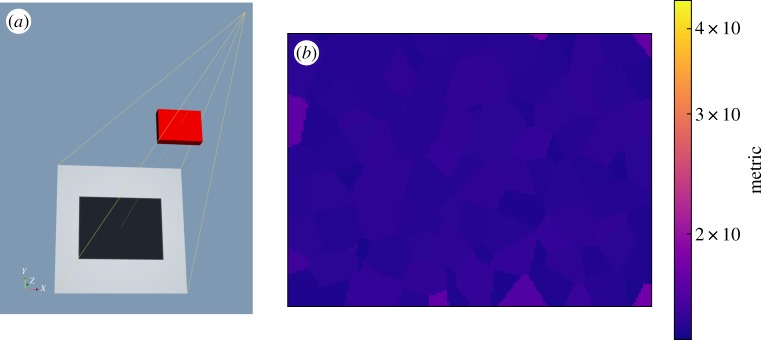
The pre-optimization state: (*a*) the configuration in aRTist is presented, (*b*) a slice through the associated detectability map is shown for the mid-plane of the block. The colour scale for the latter was set to match figure 12*b*.

Here we re-use the scenario presented in the preceding §6d, but allow all translational and rotational degrees of freedom of the sample to be optimized over (ignoring the symmetry of the sample), as a means of confirming the overall performance of the optimization for a scenario which is simple enough for a human inspector to provide a reference inspection configuration recommendation. The initial configuration is illustrated in figure 11*a*, with the corresponding detectability map shown in figure 11*b*. The map appears somewhat blotchy, as a consequence of nearest-neighbour interpolation between the 200 defect samples applied, however, the metric value is, as might be expected, quite uniform across the component volume. The overall metric value at this stage was 14.3.

**Figure 12.**
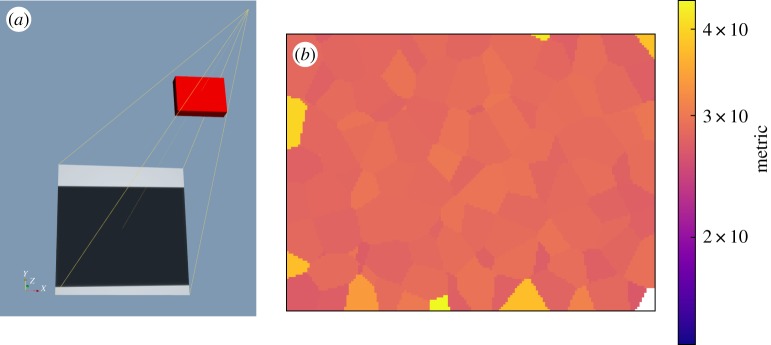
The post-optimization state: (*a*) the configuration in aRTist is presented, (*b*) a slice through the associated detectability map is shown for the mid-plane of the block. The colour scale for the latter was set to match figure 11*b*.

After 32 exploratory evolutions with a population of 24 samples, followed by eight refining evolutions with a population of 15 samples, the best solution found is illustrated in figure 12*a*. The computation time was 8.5 h on a custom workstation (AMD 1950x Threadripper CPU with 16 cores at 4 GHz, NVIDIA Titan XP GPU, 64 GB RAM), during which time 904 configurations and 180 800 defects were evaluated. The overall metric value after optimization was 26.2, indicating a notable improvement over the starting point. The detectability map in figure 12*b* further illustrates this improvement compared with figure 11*b*, and indicates the high level of uniformity retained.

The result is very reasonable by virtue of being notably similar to what a human inspector would probably suggest for this inspection scenario, maximizing the magnification as far as possible given the sample size and minimizing the in-material X-ray path length. The only apparent shortcoming of the computed configuration is that small regions at the edge of the sample are out of view. This can occur either due to an inadequate sampling of potential defects in these regions or as a consequence of the form of the objective function, where the use of the mean (across the lowest detectabilities) provides some scope for the overall metric to be increased when some defects go out of view if the remaining defects become substantially more detectable.

### (f) Full optimization trial

#### (i) Computation

In this test, we consider the test case sample geometry, and attempt to compute the optimal set of three projections. The defects selected as the targets of the inspection were 0.5 mm spherical pores, sampled uniformly across the component. 200 such defects were sampled per projection evaluated. Inspection parameters such as source voltage, current and exposure time were fixed at the same values (180 kV, 0.2 mA, 2 s, respectively) for all projections but all translational and rotational degrees of freedom were optimized over. However, only the rotation about the vertical (compatible with manipulator of a CT machine) was allowed to vary independently, and the other angles were linked across the projection set. Therefore, the dimensionality of the parameter space optimized over was 14 (table 1).

**Figure 13.**
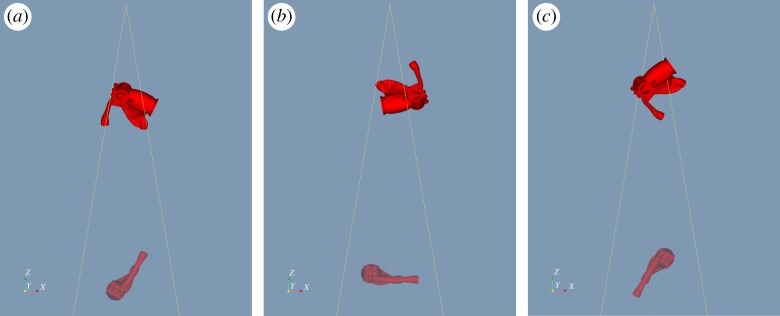
Orthographic projection aRTist scene views along the *y*-axis (corresponding to the axis of the turntable in the X-ray CT hardware) illustrating the pre- and post-optimization states of the inspection configuration. The configuration consists of three projections as shown (*a*–*c*), and each image shows both the initial state, greyed out and towards the bottom, and the final state at the top. Note that in both states the projections differ in orientation only by rotation about that axis, even after optimization, as a consequence of the linking of parameters across the projections of the projection set. (Online version in colour.)

**Figure 14.**
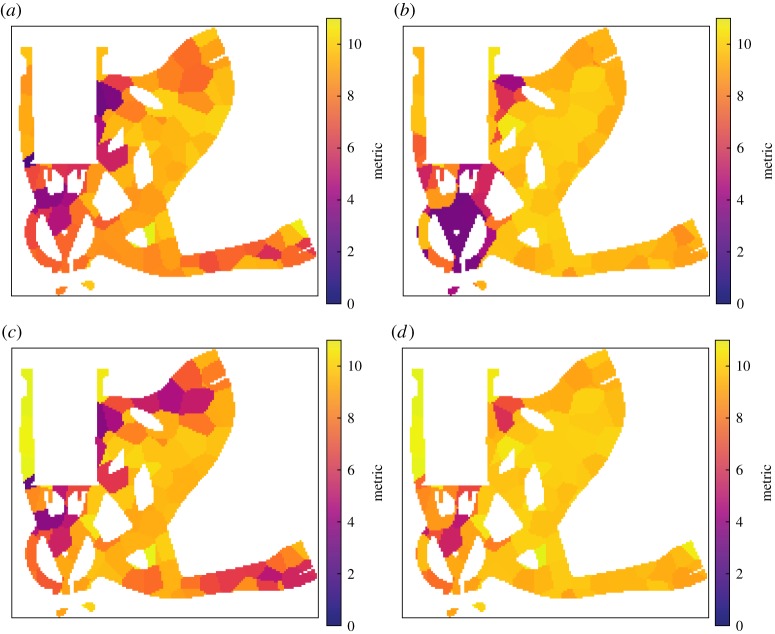
Slices through the 3D detectability maps for the component computed for the pre-optimization projections shown in figure 13. (*a*–*c*) Relate to the three projections in order, respectively, while (*d*) is the map for the overall inspection configuration, composed of the three component projections. All slices relate to the same location through the component and the colour scales were set to match.

Figure 13 illustrates the configuration the optimization was initialized with, while figure 14 provides the corresponding detectability maps, for the individual projections and the set overall. The overall metric for the configuration was determined to be 6.8.

**Figure 15.**
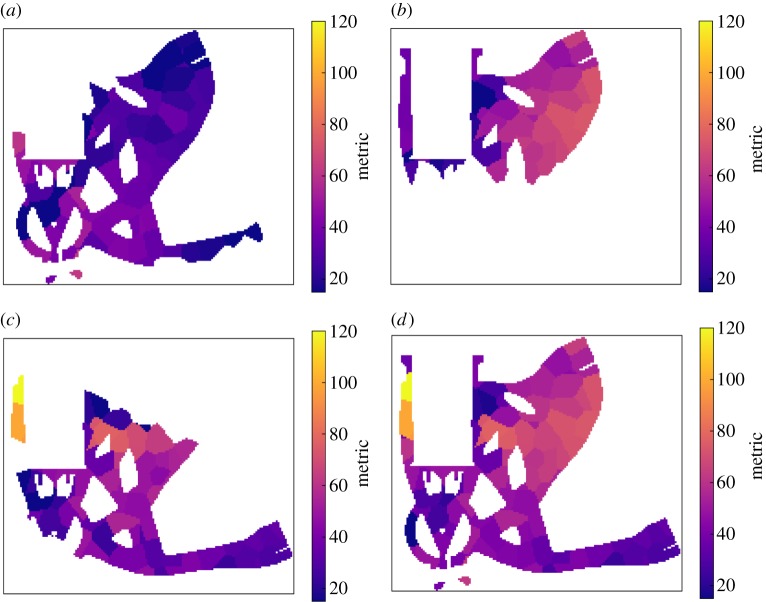
Slices through the 3D detectability maps for the component computed for the post-optimization projections shown in figure 13. (*a*–*c*) Relate to the three projections in order, respectively, while (*d*) is the map for the overall inspection configuration, composed of the three component projections. All slices relate to the same location through the component and the colour scales were set to match, but differ from figure 14 as the values here are higher throughout.

**Figure 16.**
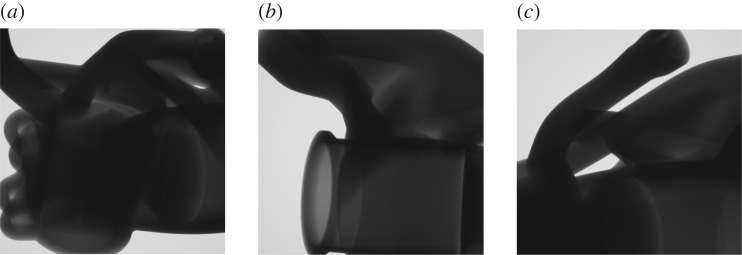
The simulated radiographs obtained for the inspection configuration suggested by the optimization.

After 24 exploratory evolutions with a population of 24 samples, followed by eight refining evolutions with a population of 15 samples, the best solution found is again illustrated in figure 13, with the corresponding detectability maps shown in figures 15. The computation time was about 39 h on the workstation described previously. A total of 3 228 555 projection sets (of three projections each) were evaluated (712 directly, 3 227 843 indirectly) in this time. The overall metric value after optimization was 24.8, indicating a substantial improvement. Note that the whole sample is not imaged in any single one of the projections, but coverage is built up over the set of projections. The simulated radiographs for the suggested projection set are illustrated in figure 16. As witnessed previously in §6e, some small elements of the geometry are not imaged in the suggested inspection configuration. It might be desirable, in practice, to adjust the form of the objective function (see §6f), and/or increase the number of defects considered, to reduce the likelihood of such behaviour.

#### (ii) Experimental demonstration

**Figure 17.**
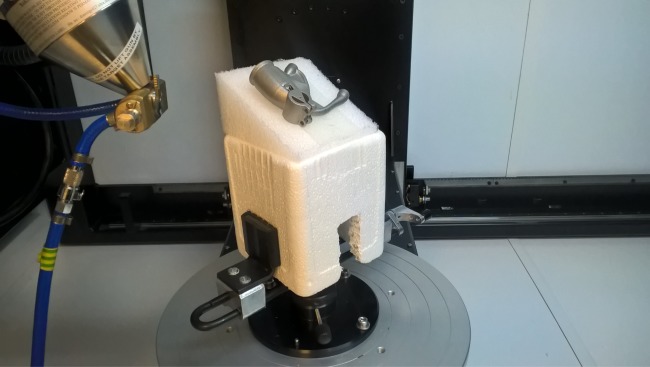
Photograph of the sample ready for practical inspection in the Nikon XTH 225 ST CT machine, with the X-ray source shown on the left. Note the angled fixturing was assembled to reflect the optimization output for the two sample orientation angles fixed across the projection set. Note also the substantial offset between the centre/origin of the sample and the origin of the turntable—an offset that must be accounted for in the hardware set-up to reproduce the configuration suggested by the simulations. (Online version in colour.)

**Figure 18.**
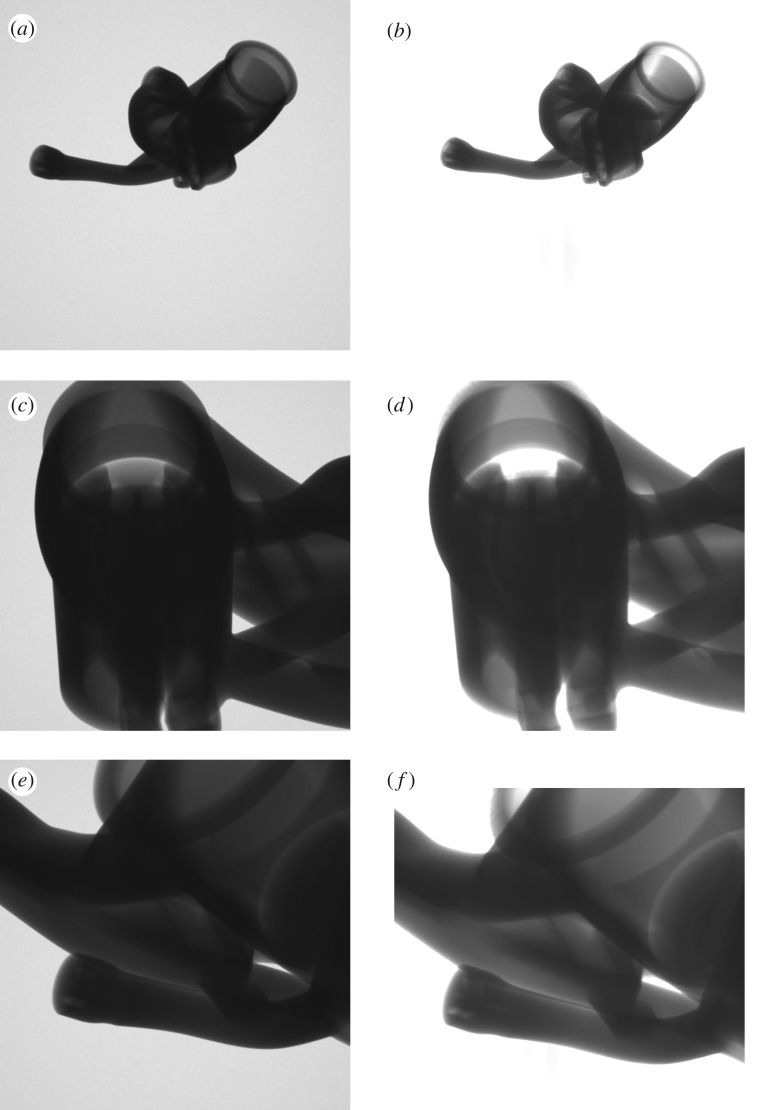
Comparison of simulated (*a*,*c*,*e*) and experimental (*b*,*d*,*f* ) radiographs for the three projections in the projection set suggested by an optimization output. The experimental images were mirrored to compensate for the discrepancy in the directionality of the output of simulated and physical detectors, but no other post-processing was applied.

An attempt was made to reproduce computed optimal projections experimentally on the physical component sample as a means of demonstrating the relevance of the computations and workflow needed to convert optimization outputs into practical data collection. To do this a fixture had to be fabricated reflecting the computed sample orientations. The physical set-up used is shown in figure 17. Experimental images were acquired using a Nikon XTH 225 ST system, based on the settings proposed by an earlier run of the algorithm for the scenario described in the preceding subsection. The coordinate systems of the CT scanner and the aRTist simulation had to be related by coordinate transforms, incorporating, for example, the origin offset associated with the fixture used. A comparison of the expected simulated and practically obtained experimental images is shown in figure 18 for all three projections in the computed projection set.

There is a good qualitative correspondence of the simulated and experimental radiographs. This clearly demonstrates the possibility of transferring insights gained from simulation to practical data acquisition. The experimental images are consistently more highly exposed than the simulated images—a consequence of the simulation model not being calibrated, and an issue to be addressed in future work.

## 7. Conclusion

This paper has presented a general framework for the formal optimization of a multi-shot imaging inspection of a complex-shaped component. The framework allows prior knowledge to be incorporated into the inspection configuration, and while it is, in principle, applicable to all image-based inspection modalities (with a suitable simulation capability), its operation has been exemplified using 2D radiography. The critical enabling innovations relate to the defect detectability evaluation for arbitrarily complex indications and the creation of an optimization algorithm with specific features for this application. Good results have been obtained for the test cases considered, and the translation of optimization outputs to experimental data collection has been demonstrated. The work therefore presents a route to achieving optimally efficient inspection and specifically reducing the reliance on X-ray CT for the inspection of complex geometries. As a useful by-product, the algorithm is able to compute the spatial distribution of a given inspection configuration’s performance. Planned future work includes the refinement of the optimization, calibration of the model and the extension of the framework to alternative inspection modalities. A further potential future direction is given by neural networks: an architecture could for example be trained on experimental outputs acquired according to the optimization, to tune the inspection configuration based on defects actually found in a set of parts of the same design.

## Supplementary Material

Experimental radiograph 1 - Fig. 18(b)

## Supplementary Material

Experimental radiograph 2 - Fig. 18(d)

## Supplementary Material

Experimental radiograph 1 - Fig. 18(f)

## Data accessibility

The experimental radiographs featured in the publication are available as electronic supplementary material.
