# Supplementary figures and images for: Optimized multi-shot imaging inspection design
Source: Proc Math Phys Eng Sci. 2018 Aug 29;474(2216):20170319. doi: 10.1098/rspa.2017.0319 (PMC6127396; doi:10.1098/rspa.2017.0319)

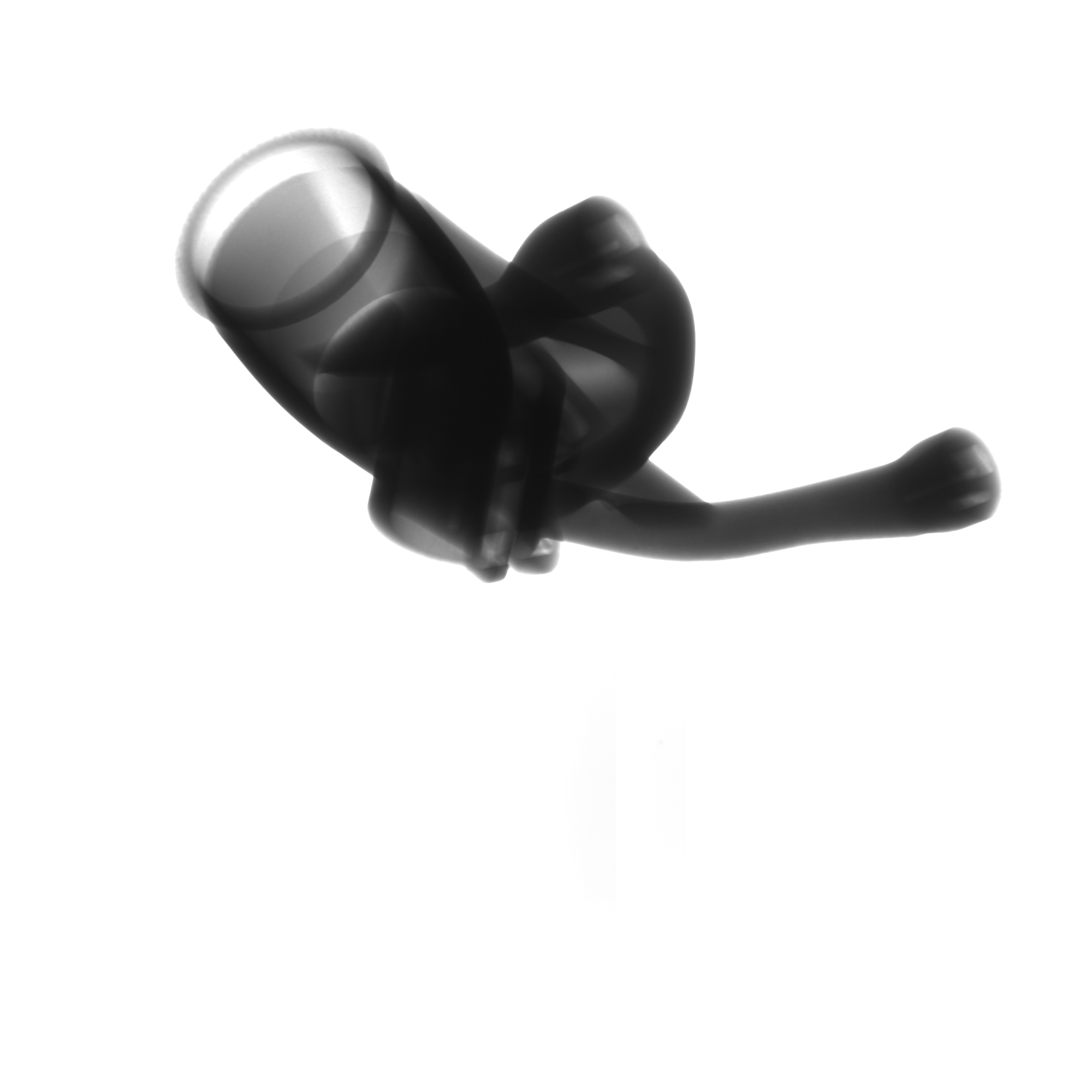

Supplement: Experimental radiograph 1 - Fig. 18(b) [file rspa20170319supp1.tif]

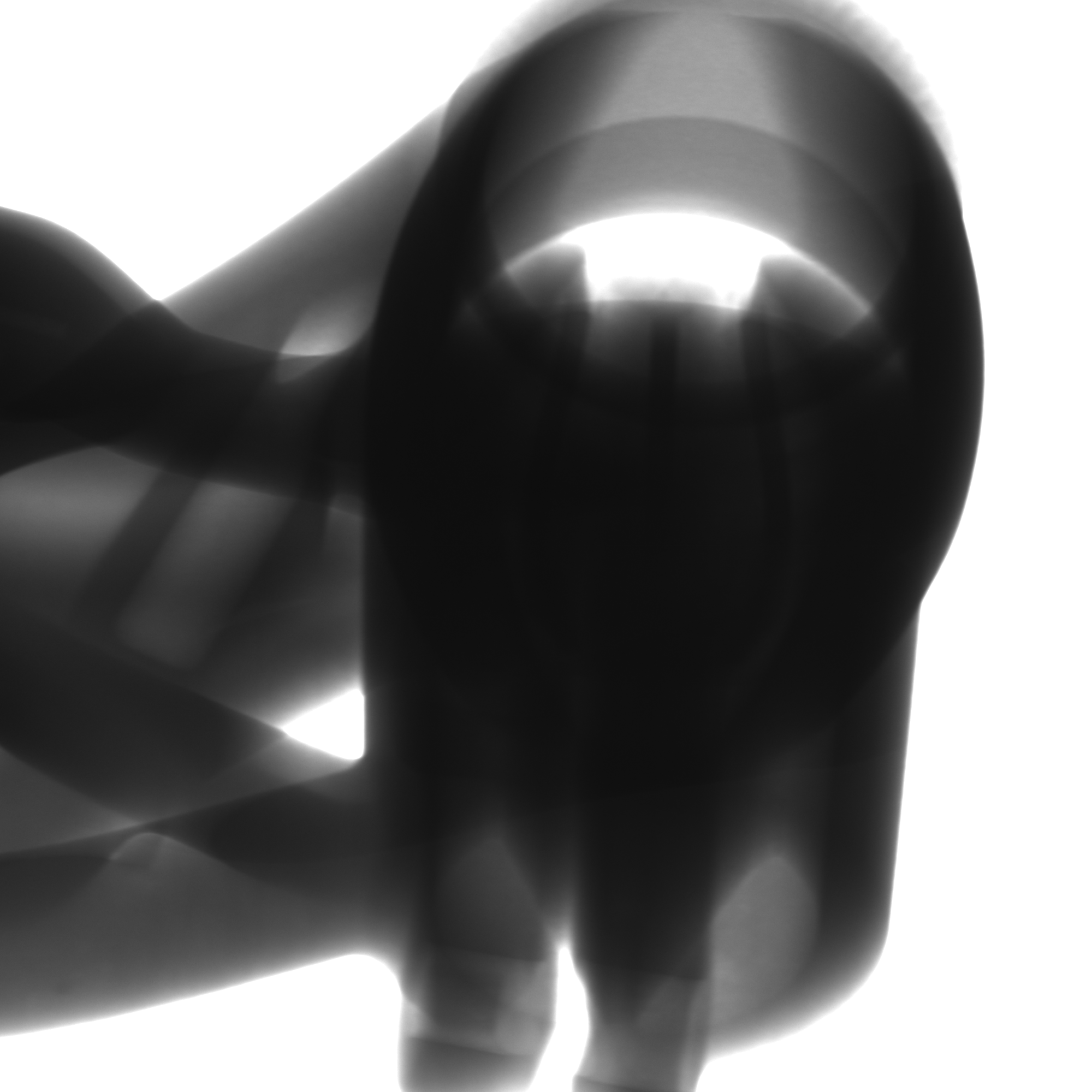

Supplement: Experimental radiograph 2 - Fig. 18(d) [file rspa20170319supp2.tif]

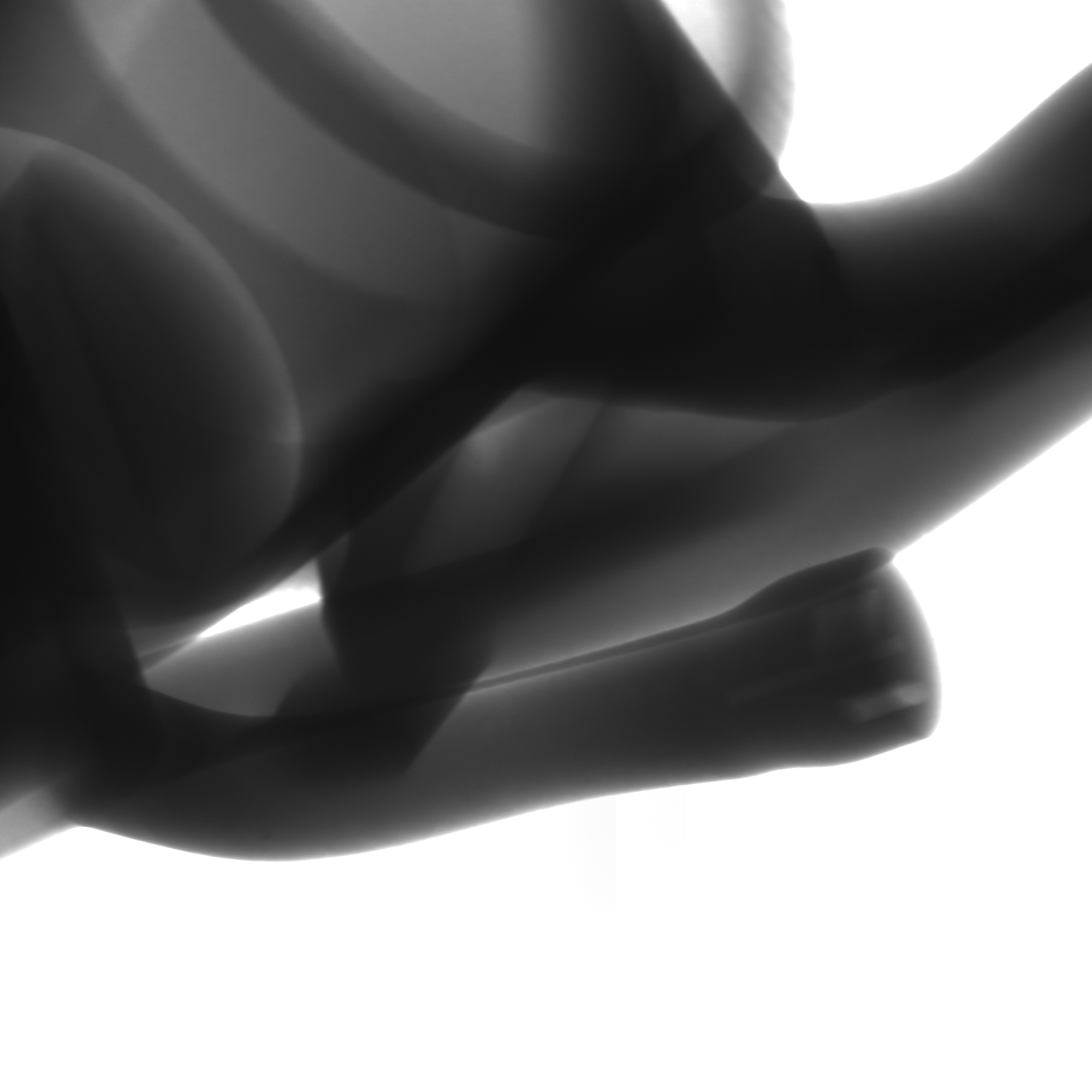

Supplement: Experimental radiograph 1 - Fig. 18(f) [file rspa20170319supp3.tif]
